# Mechanisms of chemoresistance in gastric cancer: interplay between microRNAs and the tumor microenvironment

**DOI:** 10.1007/s12094-026-04377-7

**Published:** 2026-05-06

**Authors:** Vlad-Costin Ilie, Corina-Elena Minciuna, Catalin Andras, Simona Nicoleta Turcu, Vlad Herlea, Simona Olimpia Dima, Gabriela Droc, Monica Lacatus, Stefan Tudor, Catalin Vasilescu

**Affiliations:** 1 Department of General Surgery , Fundeni Clinical Institute, Bucharest, Romania; 2 “Carol Davila” University of Medicine and Pharmacy, Bucharest, Romania; 3 Department of Pathology, Fundeni Clinical Institute, Bucharest, Romania; 4 Center of Excellence in Translational Medicine (CEMT), Fundeni Clinical Institute, Bucharest, Romania; 5 Center of Anesthesia and Intensive Care, Fundeni Clinical Institute, Bucharest, Romania

**Keywords:** Gastric cancer, Chemoresistance, microRNAs, Tumor microenvironment

## Abstract

Chemoresistance remains a major barrier to durable disease control in gastric cancer, limiting the effectiveness of perioperative and palliative systemic therapies. Increasing evidence indicates that resistance is driven by a complex interplay between tumor-intrinsic adaptations and tumor microenvironment-mediated survival pathways. Although chemoresistance can be innate or acquired, a variety of mechanisms could coexist within the tumor, with various processes acting synergistically. MicroRNAs, as key post-transcriptional regulators, have emerged as central modulators of these resistance networks. This review summarizes the principal mechanisms of chemoresistance in gastric cancer and synthesizes current evidence on how microRNAs—derived from tumor cells and the tumor microenvironment—promote or reverse resistance to commonly used chemotherapeutic agents and targeted therapy/immunotherapies, highlighting therapeutic opportunities. Evidence was organized by mechanism (drug transport and metabolism, DNA damage response, apoptosis and survival signaling, autophagy, epithelial-to-mesenchymal transition/cancer stem cell plasticity, and immune escape) and by drug class.

## Introduction

Gastric cancer (GC) remains a significant global health concern in terms of both incidence and mortality, currently ranking as the fifth leading cause of cancer-related death worldwide [1]. Despite advances in surgical techniques and the introduction of multimodal treatment strategies, chemotherapy remains a cornerstone in the management of advanced GC [2]. Perioperative chemotherapy is widely recommended for resectable advanced GC by several international guidelines, including those from NCCN, ESMO, and KGCA, aiming to enhance curative resection and control micro-metastatic disease [3–5]. In contrast, the Japanese Gastric Cancer Association (JGCA) favors postoperative adjuvant chemotherapy following D2 gastrectomy, based on strong evidence from regional clinical trials [6]. Such differences likely arise from variations in surgical approaches as well as population-specific tumor biology [7]. Furthermore, chemotherapy regimens differ significantly between Eastern and Western countries. In the West, regimens like FLOT (5-fluorouracil, leucovorin, oxaliplatin, docetaxel) are commonly used, while in Eastern Asia, S-1-based or FOLFOX/XELOX regimens are more frequently employed [3–6]. Regardless of geographic region, however, chemoresistance (CR) remains a major challenge, limiting treatment efficacy and contributing to poor outcomes in many patients [8]. CR is a multifactorial phenomenon involving both tumor-intrinsic and tumor-extrinsic mechanisms [9]. Among the key players, microRNAs (miRs) have emerged as pivotal regulators of gene expression that can alter cell survival, apoptosis, and drug response. Simultaneously, the tumor microenvironment (TME)—including stromal cells, immune infiltrates, and extracellular matrix—has gained attention as a dynamic ecosystem that modulates therapy resistance [10, 11]. Current evidence highlights a complex interplay between miRs and the TME, whereby miRs both regulate microenvironmental components and are modulated by them, contributing to an adaptive and chemoresistant tumor phenotype [12]. This review aims to explore the mechanisms of CR in GC, with a particular focus on the crosstalk between miRs and the TME, and how this interaction may offer novel therapeutic opportunities.

## ***Overview of chemoresistance in gastric cancer***

CR refers to the ability of cancer cells to evade or withstand the cytotoxic effects of chemotherapeutic agents, resulting in reduced drug efficacy and therapeutic failure [13]. It can be classified as intrinsic (innate), when present before treatment, or acquired, when it develops during or after chemotherapy exposure through adaptive cellular and molecular mechanisms. However, the variety of mechanism could coexist within the tumor, with various synergic processes [14]. Therefore, CR significantly compromises the efficacy of systemic therapies and is closely linked to tumor progression, metastasis, and recurrence, being responsible for up to 90% of the cancer-related deaths [15].

The molecular heterogeneity of GC, both between patients and within individual tumors, contributes to widely variable responses to chemotherapy regimens. Current data indicates that more than 50% of GC patients exhibit intrinsic or acquired resistance to chemotherapy, which contributes to poor 5 year survival rates and represents a major challenge in the management of this disease [16].

First-line treatment for advanced GC typically involves combination chemotherapy, with regimens selected based on patient performance status, HER2 status, and regional guidelines. Since the results shown by the FLOT4-AIO trial in 2019 [17], the standard first-line therapy in Western countries is the FLOT regimen (5-fluorouracil, leucovorin, oxaliplatin, and docetaxel), especially in the perioperative setting for resectable disease. For patients unfit for triplet chemotherapy, a combination of a fluoropyrimidine plus platinum-based doublets is recommended (capecitabine or 5-fluorouracil combined with cisplatin or oxaliplatin) [3, 4]. Additionally, targeted therapy is recommended in HER2 overexpression, the addition of trastuzumab being the standard of care based on results from the pivotal ToGA trial [18]. A checkpoint inhibitor (nivolumab or pembrolizumab) should be added to first-line chemotherapy for patients with advanced disease with PD-L1 and MSI-H/dMMR tumors [19, 20].

In Eastern countries, particularly Japan and Korea, S-1-based regimens (S-1 and docetaxel or capecitabine plus oxaliplatin) are commonly used and have demonstrated non-inferiority to Western standards in region-specific trials [21, 22].

The most used second-line agents include irinotecan, docetaxel, and paclitaxel, either as monotherapy or in combination with ramucirumab, a monoclonal antibody targeting VEGFR-2 (antiangiogenic effect) [23]. The RAINBOW trial demonstrated that paclitaxel plus ramucirumab significantly improved overall survival compared to paclitaxel alone, establishing this combination as a preferred second-line option for HER2-negative disease [23].

Anthracyclines, such as doxorubicin and epirubicin, have been used in the treatment of advanced GC, particularly in combination regimens. The ECF regimen (epirubicin, cisplatin, and 5-fluorouracil) was a widely used first-line therapy in Western countries for many years and demonstrated moderate efficacy [24], leading to the gradual replacement by newer combinations like FLOT. Today, anthracyclines are largely considered legacy agents in GC treatment, with their use reserved for specific clinical contexts or in regions where newer regimens are not widely available.

Despite the development of multiple systemic and targeted therapeutic strategies, clinical outcomes remain strongly influenced by the emergence of CR. A deeper understanding of the molecular mechanisms driving resistance is therefore essential to improve treatment efficacy in GC.

## Mechanisms of chemoresistance in GC

The mechanisms of CR involved in GC are multifaceted [9, 25]:

- Altered intracellular drug availability, driven by decreased expression of membrane uptake transporters and/or increased activity of drug efflux pumps
- Impaired prodrug activation or increased drug inactivation, due to changes in metabolic and detoxification enzyme expression or activity
- Altered expression or function of molecular targets
- Enhanced DNA repair mechanisms
- Inhibition of apoptosis and activation of pro-survival pathways
- Epithelial-to-mesenchymal transition (EMT) and stemness programs
- Reinforcement of autophagy
- Interaction of the tumor cell with the microenvironment

All the mechanisms are synthetized in Table 1, with the key genes/molecules and the main affected drugs involved.

**Table 1:**
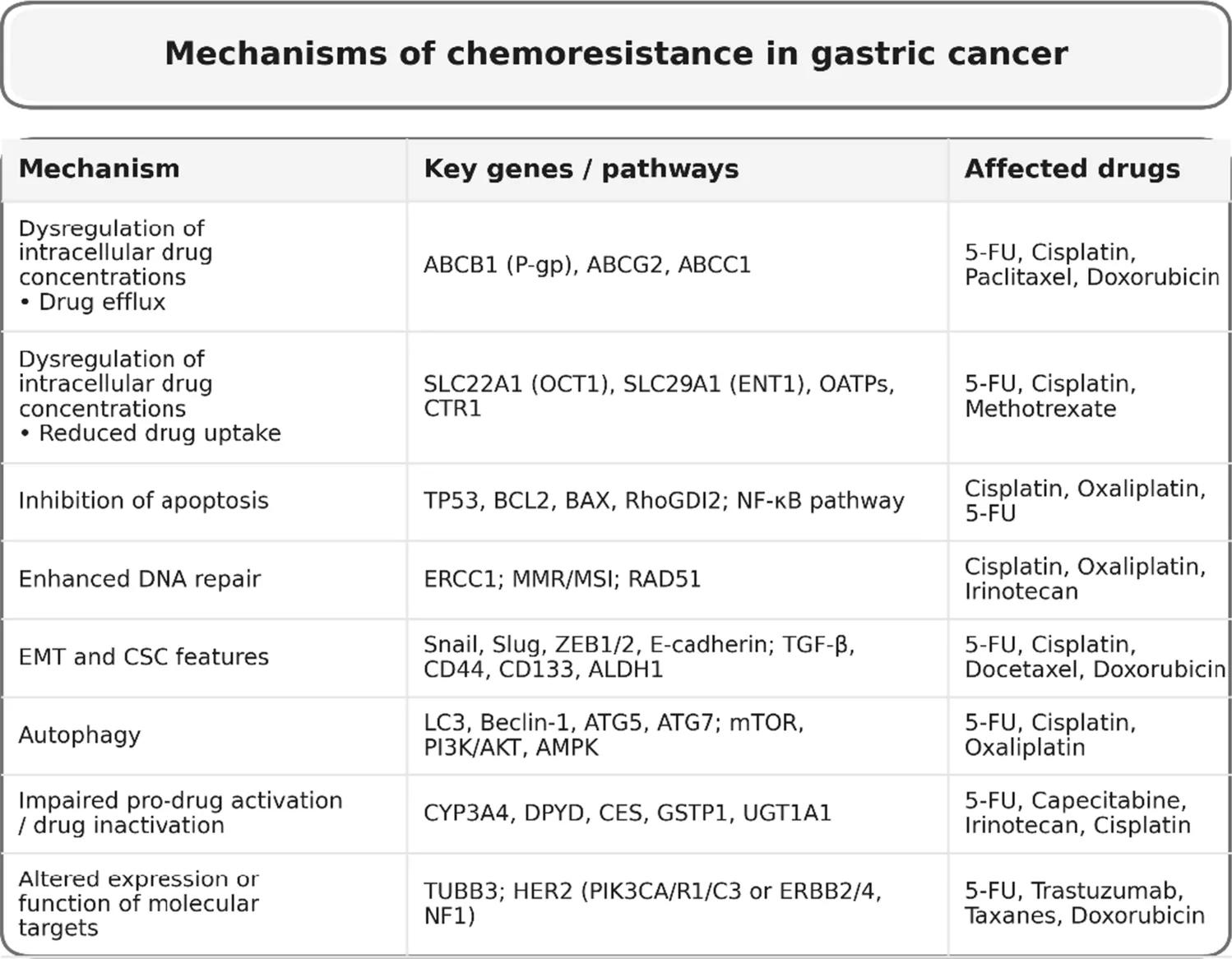
Mechanisms of CR involved in GC

### Dysregulation of membrane transporters: impaired drug uptake and enhanced efflux

Dysregulation of membrane transporters represents a major mechanism of CR in GC by altering intracellular drug bioavailability through both impaired influx and enhanced efflux.

On the one hand, reduced expression of membrane uptake transporters limits intracellular accumulation of chemotherapeutic agents. Key solute carrier (SLC) families are implicated in this process. Organic cation transporters (OCTs) mediate the uptake of drugs, such as anthracyclines, platinum derivatives, and irinotecan, while organic anion transporting polypeptides (OATPs) facilitate the entry of irinotecan and docetaxel [26]. Similarly, the copper transporter CTR1, which is essential for cisplatin uptake, is frequently downregulated in resistant tumors [26]. Nucleoside transporters, including concentrative nucleoside transporters (CNTs) and equilibrative nucleoside transporters (ENTs), are involved in the uptake of 5-fluorouracil (5-FU) and are often suppressed in chemoresistant cancer cells [26].

On the other hand, overexpression of drug efflux pumps, particularly members of the ATP-binding cassette (ABC) transporter family, plays a critical role in mediating multidrug resistance. The ABCB1 gene (also known as MDR1 or P-glycoprotein), part of the B subfamily, is upregulated in response to anthracyclines and docetaxel exposure [26]. The multidrug resistance-associated proteins (MRPs), encoded by members of the ABCC subfamily, are known to mediate efflux of anthracyclines, platinum agents, 5-FU, and taxanes. ABCG2, encoding the breast cancer resistance protein (BCRP), is similarly associated with efflux of anthracyclines and 5-FU. Additionally, P-type ATPases, such as ATP7A and ATP7B, which mediate copper homeostasis, have been implicated in resistance to platinum-based compounds by exporting intracellular cisplatin and reducing its cytotoxic activity [26]. Together, these alterations in drug transporter expression substantially reduce intracellular drug concentrations, thereby limiting therapeutic efficacy.

### Inhibition of apoptosis and activation of survival pathways

Evasion of apoptosis, or programmed cell death, is a well-recognized mechanism of CR in GC. Numerous pro-apoptotic and anti-apoptotic signaling proteins are disrupted in resistant tumors, leading to impaired therapeutic efficacy of agents, such as 5-FU, cisplatin, and taxanes.

Tumor protein p53, a key regulator of the DNA damage response and apoptosis, is one of the most frequently mutated genes in GC. Wild-type p53 functions as a pro-apoptotic tumor suppressor, and its activity is associated with increased sensitivity to DNA-damaging agents. Clinical studies have demonstrated that p53 status may serve as a predictive biomarker for response to chemotherapy [27]. In contrast, mutations in the TP53 gene lead to loss of p53 function, impairing apoptotic responses and promoting resistance to 5-FU and cisplatin [28]. Restoration of p53 function in resistant cells has been shown to resensitize tumors to cisplatin via inhibition of the Akt survival pathway, supporting the potential of p53-based therapeutic strategies [29].

The BCL-2 protein family, which governs mitochondrial outer membrane permeabilization, plays a pivotal role in determining the apoptotic threshold. Overexpression of anti-apoptotic proteins such as BCL-2 correlates with resistance to 5-FU [30]. Similarly, Rho GDP dissociation inhibitor 2 (RhoGDI2) enhances resistance to cisplatin by upregulating BCL-2 expression in GC cells, as demonstrated in overexpressing SNU-484 cell lines [31]. Other mitochondrial apoptotic regulators, including BAK and BAX, are also frequently suppressed in resistant tumors. Low expression of BAK has been associated with docetaxel resistance [32], while reduced BAX levels correlate with poor prognosis in patients treated with FOLFOX (5-FU, leucovorin, and oxaliplatin) [33].

In addition to inhibition of pro-apoptotic proteins, gastric tumors may activate compensatory pro-survival signaling pathways, most notably the nuclear factor-kappa B (NF-κB) pathway. NF-κB promotes transcription of several anti-apoptotic genes, including survivin, BCL-XL, and XIAP, thereby enhancing cell survival in the presence of chemotherapy. For instance, cancer-associated fibroblast (CAF)-derived interleukin-8 (IL-8) has been shown to induce cisplatin resistance via NF-κB activation and ABCB1 upregulation [34]. Moreover, pharmacologic agents such as simvastatin have demonstrated the potential to reverse this resistance by suppressing NF-κB-regulated survival markers, thereby enhancing the cytotoxicity of capecitabine [35].

Together, these findings emphasize the central role of apoptosis dysregulation and survival pathway activation in GC CR and point to potential molecular targets for therapeutic intervention.

### Enhanced DNA repair capacity

An important contributor to CR is the upregulation or dysregulation of DNA repair mechanisms, which allows tumor cells to efficiently repair cytotoxic damage induced by chemotherapy, particularly DNA-damaging agents like platinum compounds and fluoropyrimidines.

One of the key players in this process is excision repair cross-complementing gene 1 (ERCC1), a crucial component of the nucleotide excision repair (NER) pathway. Overexpression of ERCC1 enhances the repair of DNA adducts formed by platinum compounds, such as cisplatin and oxaliplatin, leading to resistance. Clinical studies have shown that patients with ERCC1-positive tumors exhibit poor responses to platinum-based chemotherapy, and it has been suggested that alternative regimens should be considered in this subgroup [36].

Another central DNA repair pathway involved in CR is the mismatch repair (MMR) system, responsible for correcting base–base mismatches and insertion–deletion loops during DNA replication. Key MMR proteins include MLH1, PMS2, and PMS1. Deficiencies in MMR proteins, particularly through loss of MLH1 expression, result in microsatellite instability (MSI)—a hypermutator phenotype observed in a subset of gastric cancers [37]. MSI status has dual implications: while it is associated with reduced responsiveness to 5-FU-based chemotherapy, it may also predict sensitivity to immune checkpoint inhibitors [37, 38]. Therefore, MSI and MMR protein expression are valuable predictive biomarkers for guiding chemotherapy selection.

Additionally, homologous recombination repair (HRR) mechanisms, particularly those involving RAD51, contribute to CR. Overexpression of RAD51, a key recombinase facilitating error-free repair of DNA double-strand breaks, has been associated with resistance to 5-FU and platinum agents [39]. In contrast, IRF1 (interferon regulatory factor 1), a tumor suppressor, has been shown to downregulate RAD51 expression, enhancing chemotherapy-induced cytotoxicity. This suggests that targeting RAD51 or restoring IRF1 function could represent promising strategies to overcome DNA repair-mediated resistance in GC [39].

### Epithelial-to-mesenchymal transition (EMT)

Epithelial-to-mesenchymal transition (EMT) is a dynamic cellular reprogramming process in which epithelial cells lose their characteristic polarity and cell–cell adhesion, while acquiring mesenchymal traits, such as motility, invasiveness, and resistance to apoptosis. Although EMT is essential in physiological contexts like embryogenesis and wound healing, it is frequently co-opted in cancer to drive tumor progression, metastasis, and therapeutic resistance [40].

In GC, EMT is closely linked to the development and maintenance of cancer stem cell (CSC) phenotypes, which are characterized by a quiescent or slow-cycling state and thus evade cytotoxic effects of conventional chemotherapy. Drugs, such as 5-FU, cisplatin, and taxanes, are primarily effective against proliferating cells and have limited efficacy against EMT-induced CSC populations [41]. EMT in gastric tumors is orchestrated by multiple signaling axes, including transforming growth factor-beta (TGF-β), and is regulated by a network of transcription factors (TFs), such as Snail, Slug, and ZEB1/2, non-coding RNAs, and stemness-associated proteins [42–44].

TGF-β signaling plays a pivotal role in inducing EMT, as demonstrated by the upregulation of mesenchymal markers vimentin and N-cadherin, which correlate with diminished sensitivity to 5-FU in gastric adenocarcinoma models [45]. Cell surface proteins, such as CD44 and CD133, are also widely expressed on gastric CSCs and have been implicated in drug resistance. CD44 overexpression has been linked to resistance to both 5-FU and etoposide, and is considered a potential prognostic biomarker in GC [46]. Similarly, CD133 expression has been associated with CR to 5-FU/cisplatin-based regimens [47].

Enzymatic markers such as aldehyde dehydrogenase 1 (ALDH1), which is active in CSCs, further contribute to resistance. Elevated ALDH1 activity has been correlated with resistance to 5-FU, supporting its use as a prognostic indicator and therapeutic target [48]. Mechanistic studies have shown that overexpression of EMT transcription factors, particularly Snail, increases the population of ALDH1-positive circulating tumor cells (CTCs). Moreover, co-expression of Snail, ZEB1, and CD44 in tumor tissue has been associated with poor clinical outcomes and early recurrence, further emphasizing the role of EMT-CSC plasticity in CR [49, 50].

These findings underscore the clinical significance of EMT in conferring drug resistance in GC and highlight the therapeutic potential of EMT and CSC-targeted strategies to enhance the efficacy of current treatment regimens.

### Reinforcement of autophagy

Autophagy is a highly conserved, lysosome-dependent catabolic process that degrades and recycles intracellular components to maintain cellular homeostasis. In the context of cancer therapy, autophagy plays a complex and dualistic role, acting either as a mechanism of cell death or as a survival strategy that enables tumor cells to withstand cytotoxic stress. In GC, this process often becomes cytoprotective in response to chemotherapy, contributing to the development of CR.

The autophagic response is orchestrated by a series of autophagy-related genes (ATGs), including Beclin-1, LC3B, ATG5, and ATG7, and is regulated upstream by signaling pathways, such as PI3K/AKT/mTOR and AMP-activated protein kinase (AMPK) [51]. Dysregulation of these pathways—particularly suppression of mTOR signaling or activation of AMPK has been shown to facilitate autophagy induction under chemotherapy-induced stress [52]. In vitro and in vivo studies have demonstrated that exposure to agents, such as 5-FU, cisplatin, and oxaliplatin, activates autophagy as a survival mechanism in cancer cells, thereby attenuating drug-induced apoptosis and promoting resistance [51–53].

Furthermore, pharmacological or genetic inhibition of autophagy-related genes (e.g., ATG5 or Beclin-1) has been shown to restore chemosensitivity, supporting the hypothesis that autophagy acts predominantly as a protective mechanism in the context of GC treatment [51, 52]. These findings suggest that combining chemotherapeutic agents with autophagy inhibitors (such as chloroquine or hydroxychloroquine) may represent a promising therapeutic strategy to overcome resistance.

### Impaired drug activation and metabolic inactivation

CR in GC can also result from alterations in the metabolism of chemotherapeutic agents, particularly through the impaired activation of pro-drugs or the enhanced inactivation of active drugs. Several metabolic enzymes are involved in these processes, influencing both efficacy and toxicity.

Members of the cytochrome P450 (CYP) enzyme family, especially CYP2A6, play a dual role in the metabolism of anticancer agents: they are responsible for both detoxification and bioactivation of pro-drugs. CYP2A6 contributes to the metabolic activation of 5-FU; genetic polymorphisms in this enzyme have been associated with altered drug efficacy and poorer clinical outcomes [54, 55]. Similarly, dihydropyrimidine dehydrogenase (DPYD), the rate-limiting enzyme in 5-FU catabolism, influences drug availability and sensitivity. High DPYD activity leads to rapid degradation of 5-FU, reducing its cytotoxic effects and contributing to therapeutic resistance [56].

In the case of irinotecan, a pro-drug requiring enzymatic conversion to its active metabolite SN-38, carboxylesterases (CES), plays a critical role. Variability in CES activity has been shown to predict therapeutic response and resistance to irinotecan-based regimens [57]. Furthermore, detoxification enzymes such as glutathione *S*-transferases (GSTs), particularly the GSTP1 isoform, are frequently overexpressed in resistant gastric tumors. This overexpression enhances conjugation and elimination of drugs like 5-FU and cisplatin, thus diminishing their intracellular cytotoxic potential [58].

In addition, UDP-glucuronosyltransferases (UGTs)—especially the UGT1A family—are essential for the glucuronidation and inactivation of SN-38. Polymorphisms or upregulation of UGT1A1, in particular, have been linked to irinotecan resistance by increasing drug clearance and reducing effective concentrations of the active metabolite [59]. Collectively, these metabolic alterations represent a critical mechanism through which GC cancer cells evade the cytotoxic effects of chemotherapy.

### Altered expression or function of molecular targets

The efficacy of chemotherapeutic and targeted agents often depends on the expression and integrity of specific molecular targets. Consequently, genetic alterations or aberrant expression of these targets can directly mediate both innate and acquired resistance.

A prominent example is the HER2 receptor (encoded by *ERBB2*), a well-established target in HER2-positive GC. Primary or acquired resistance to trastuzumab—an anti-HER2 monoclonal antibody—has been associated with mutations not only in ERBB2, but also in downstream signaling components, such as PIK3CA, PIK3R1, and PIK3C3 [60]. Notably, mutations in ERBB4, such as S774G variant, may paradoxically enhance sensitivity to HER2-targeted therapy, suggesting potential avenues for overcoming resistance [60]. In the context of Epstein–Barr virus (EBV)-positive GC, which represents a distinct molecular subtype, frequent PIK3CA mutations may contribute to trastuzumab resistance and require further therapeutic stratification [61]. Additionally, mutations or loss of NF1—a tumor suppressor that negatively regulates RAS signaling—have also been linked to resistance to HER2 blockade [60].

Regarding anti-angiogenic therapy, no validated predictive biomarkers have been identified for ramucirumab, a VEGFR2-targeting monoclonal antibody. However, exploratory analyses have suggested that high VEGFR2 expression on tumor endothelium may be associated with a trend toward shorter progression-free survival, although this finding did not reach statistical significance [62, 63].

Resistance to taxane-based chemotherapy, including docetaxel and paclitaxel, has been associated with aberrant expression of β-tubulin isotypes, particularly class III β-tubulin (TUBB3). This microtubule protein, normally expressed in neuronal tissues, disrupts microtubule dynamics when ectopically expressed in tumors and has been implicated in resistance to both docetaxel [64] and paclitaxel [65].

Together, these alterations in target molecules or their associated signaling networks limit the efficacy of both cytotoxic and targeted therapies, highlighting the need for personalized molecular profiling in treatment planning for GC.

## Role of microRNAs in chemoresistance

MicroRNAs (miRs) are small, non-coding RNAs (approximately 18–25 nucleotides in length) that regulate gene expression post-transcriptionally by binding to complementary sequences in the 3′ untranslated regions (UTRs) of target mRNAs, leading to mRNA degradation or translation inhibition [66]. In GC, dysregulation of specific miRs has been strongly associated with the development of CR through a variety of mechanisms, including modulation of apoptosis, drug transport, EMT, autophagy, and the DNA damage response.

### miRs regulating drug efflux and drug uptake

**Fig. 1:**
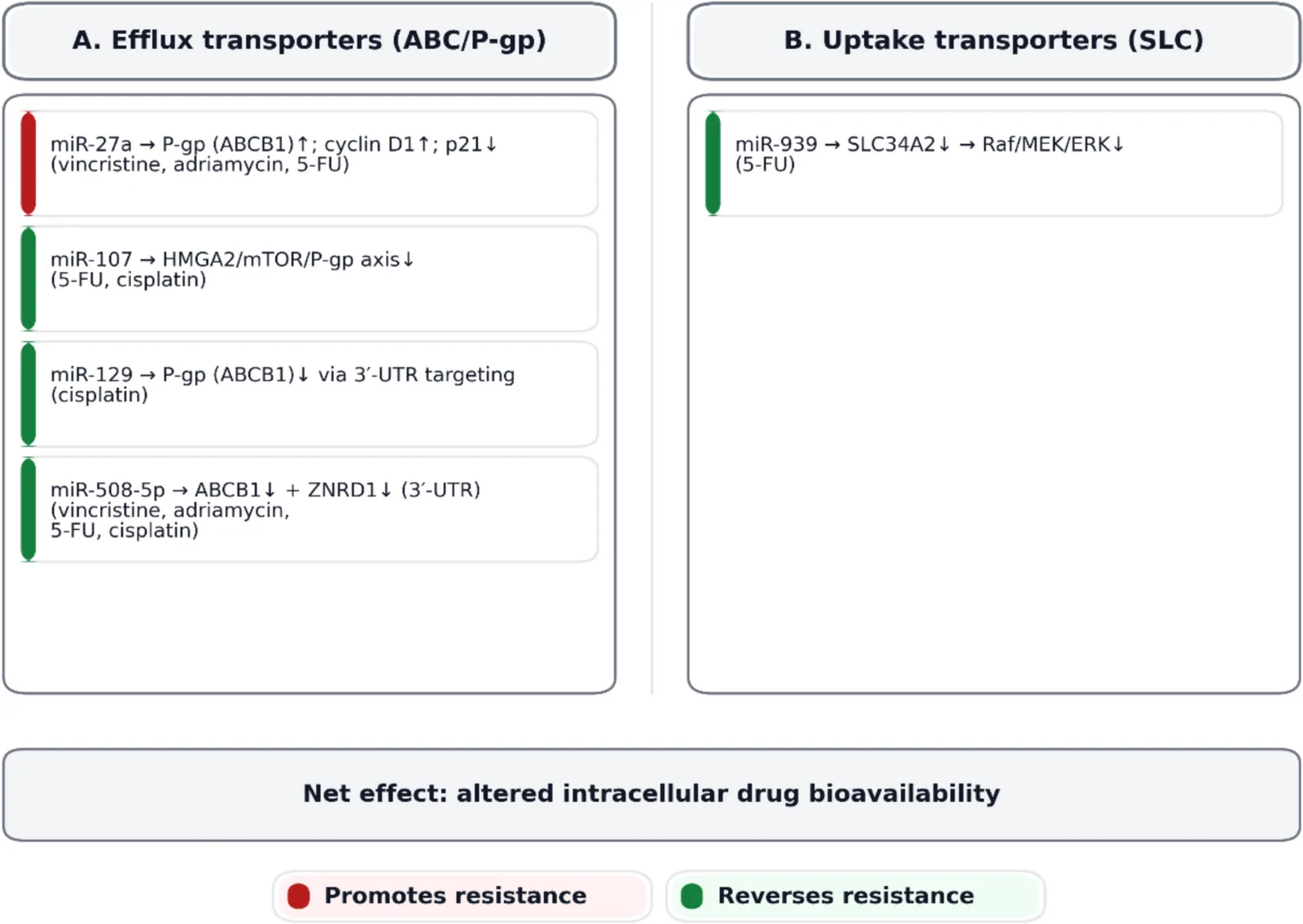
miRs involved in regulation of membrane transporters, affecting the intracellular drug bioavailability through both impaired uptake and efflux. Chemosensitizing miRs are indicated in green, whereas resistance-promoting miR is shown in red

One of the most studied CR mechanisms is overexpression of ATP-binding cassette (ABC) family proteins, efflux transporters which reduce intracellular drug accumulation by pumping drugs out of cancer cells. Several miRs that regulate these transporters might be considered as useful targets for GC therapy (Fig. 1):

- miR-27a can increase the expression of P-glycoprotein and the activity of cyclin D1, while down-regulating the expression of p21, thereby promoting resistance to vincristine, adriamycin, and 5-FU [67].
- miR-107 enhances the sensitivity of resistant tumor cells to chemotherapy by down-regulating the HMGA2/mTOR/P-gp axis, thereby reversing resistance to 5-FU and cisplatin [68].
- miR-129 could also reverse the cisplatin resistance in GC cells, inhibiting P-gp expression by directly targeting its 3′-UTR [69].
- miR-508—5p could also target the 3′-UTR of ABCB1 and Zinc ribbon domain-containing 1 (ZNRD1), reversing cancer cell resistance to multiple chemotherapeutics in vitro and sensitize tumors to chemotherapy in vivo, such as vincristine, adriamycin, 5-FU, and cisplatin [70].

Beyond efflux-mediated resistance, miRs may also influence drug uptake, primarily through modulation of solute carrier (SLC) transporters, although these mechanisms are less extensively characterized in GC (Fig. 1):

- miR-939 could suppress GC cell growth and enhance 5-FU-induced chemosensitivity through inhibiting SLC34A2/Raf/MEK/ERK pathway [71].

### miRs and apoptosis inhibition

**Fig. 2:**
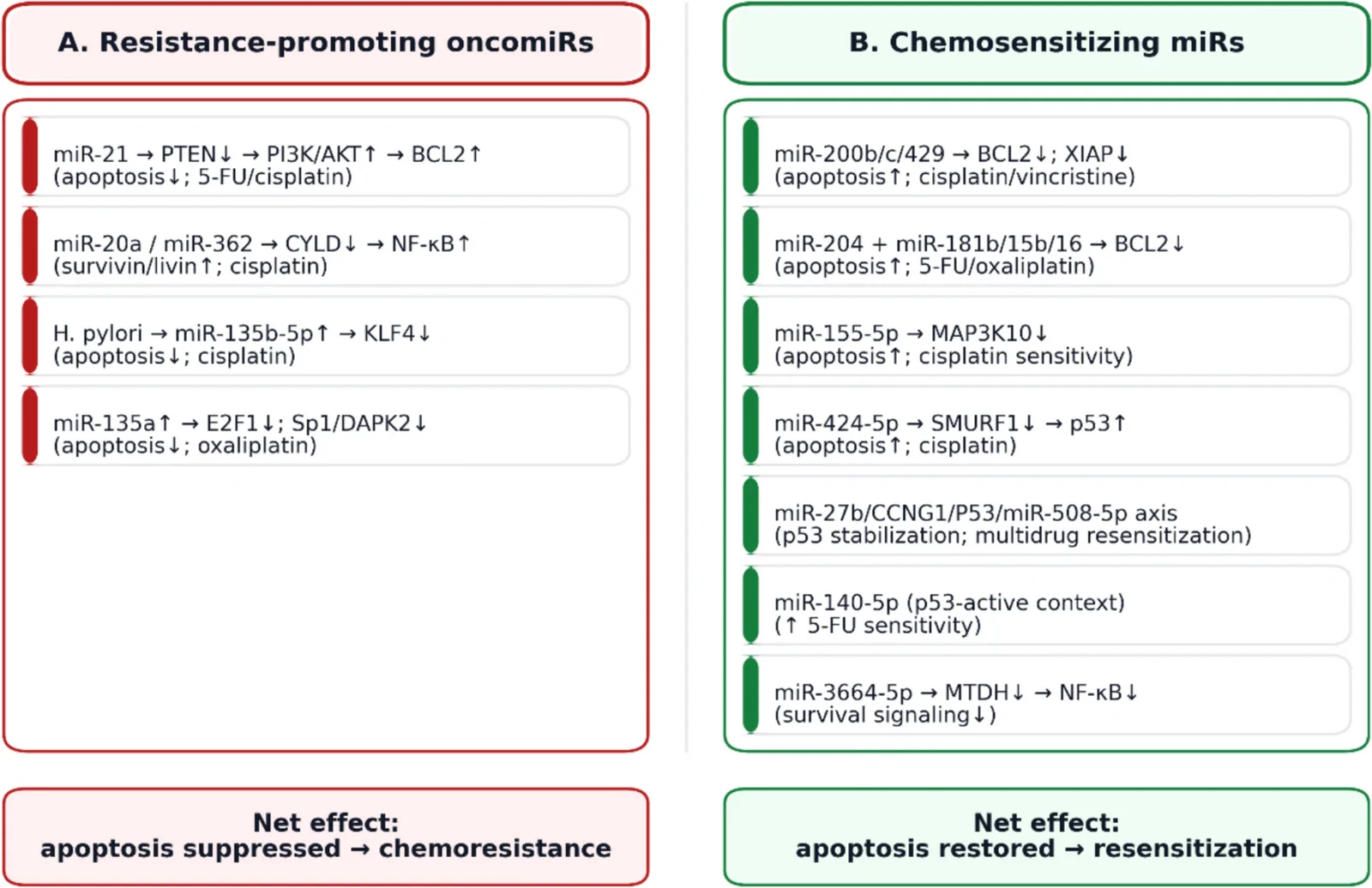
Apoptosis-related miRs. Chemosensitizing miRs are indicated in green, whereas resistance-promoting miRs are shown in red

Dysregulated miRs contribute to CR by shifting the balance between pro- and anti-apoptotic signaling, as shown in Fig. 2.

OncomiRs that are overexpressed in GC can promote resistance by suppressing apoptotic effectors or activating pro-survival pathways:

- miR-21 targets the tumor suppressor PTEN, leading to hyperactivation of the PI3K/Akt pathway and increased expression of the anti-apoptotic protein BCL-2, thereby reducing sensitivity to 5-FU and cisplatin [72].
- miR-20a and miR-362 can directly repress the expression of cylindromatosis (CYLD), resulting in NF-κB activation and upregulation of downstream anti-apoptotic mediators, such as survivin and livin, which may contribute to cisplatin resistance [73, 74].
- *Helicobacter pylori* infection may induce the expression of miR-135b-5p, mediated by the NFκB pathway. Thereby, miR-135b-5p targets Krüppel-like factor 4 (KLF4), leading to reduced KLF4 expression, suppressing apoptosis and inducing cisplatin resistance [75].
- miR-135a promotes GC pathogenesis and resistance to oxaliplatin by suppressing the E2F1 expression and Sp1/DAPK2 pathway signaling [76].

Conversely, tumor-suppressive miRs can restore chemosensitivity by reactivating p53 signaling, inhibiting BCL-2 family members, or dampening NF-κB–dependent survival programs:

- miR-200b/c/429 cluster has been shown to regulate apoptotic susceptibility by targeting anti-apoptotic molecules, such as BCL2 and XIAP, thereby modulating sensitivity to agents including cisplatin and vincristine [77].
- miR-204 targeted Bcl-2 messenger RNA and increased responsiveness of GC cells to 5-FU and oxaliplatin treatment [78]. Similar chemosensitizing effects through BCL-2 suppression have been described for miR-181b, miR-15b, and miR-16 [79, 80].
- miR-155-5p targets MAP3K10 and promotes apoptosis, inhibiting gastric cell proliferation. Overexpression of miR-155-5p may increase the cisplatin sensitivity of tumor cells [81].
- miR-27b directly targets the 3'-UTR regions of CCNG1, a well-known negative regulator of P53 stability, a master tumor suppressor that has been shown to directly regulate miR-508-5p. Thus, the miR-27b/CCNG1/P53/miR-508-5p axis may play an important role in GC-associated CR, sensitizing tumor cells to several chemotherapeutic agents like adriamycin, vincristine, 5-FU and cisplatin [82].
- Overexpression of mir-424-5p downregulates the expression of SMURF1, which may increase p53 expression, promoting apoptosis and the sensitivity of GC cells to cisplatin treatment [83, 84].
- miR-140-5p appears to act in a p53-dependent context; its upregulation in tumors with activated p53 has been associated with increased sensitivity to 5-FU and improved outcomes, particularly in intestinal-type GC [85].
- miR-3664-5P suppresses the proliferation and metastasis of GC cells by attenuating the NF-κB signaling pathway via metadherin MTDH targeting and may have a role in CR [86].

### miRs modulating DNA damage response

**Fig. 3:**
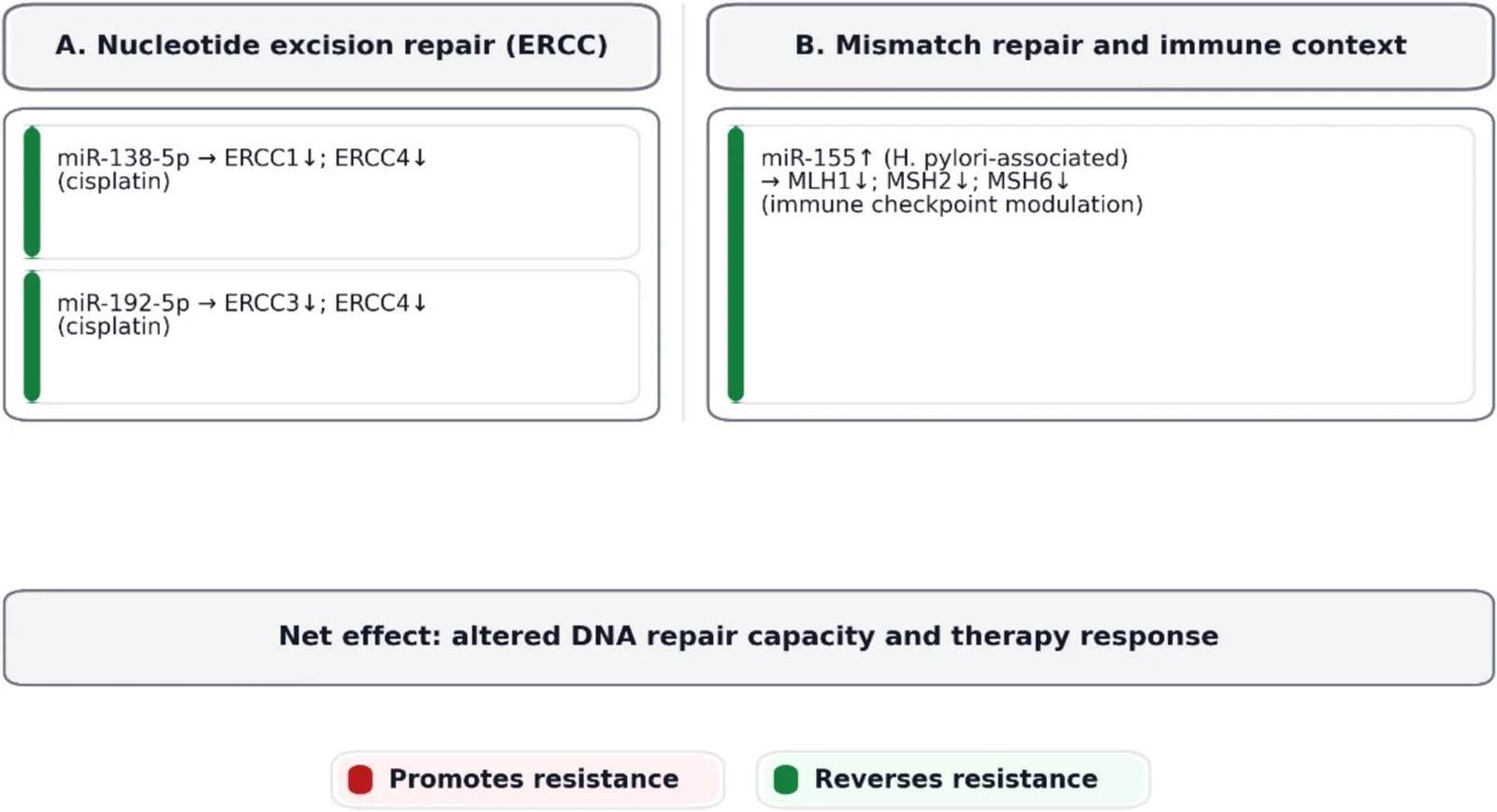
Sensitizing miRs modulating DNA damage response are indicated in green

Several miRs can influence DNA repair capacity, thus affecting the response to chemotherapeutic agents, as shown in Fig. 3:

- miR-138-5p modulates the expression of DNA repair proteins ERCC1 and ERCC4 and could reverse the cisplatin resistance in GC [87].
- miR-192-5p may also increase the sensitivity to cisplatin, by inhibiting the expression levels of ERCC3 and ERCC4, and could represent a potential biomarker and therapeutic target in cisplatin resistance [88].
- miR-155 is considered a key marker of chronic gastric inflammation that predisposes a patient to gastric carcinogenesis, his expression being observed after *Helicobacter pylori* infection. Overexpression of miR-155 downregulates the expression of certain MMR genes, such as MLH1, MSH2, and MSH6, and may have a role in the modulation of immune checkpoint regulation. These data suggest that the augmentation of miR-155 expression could potentially improve anticancer immunotherapies [89].

### miRs influencing EMT and cancer stem cell features

**Fig. 4:**
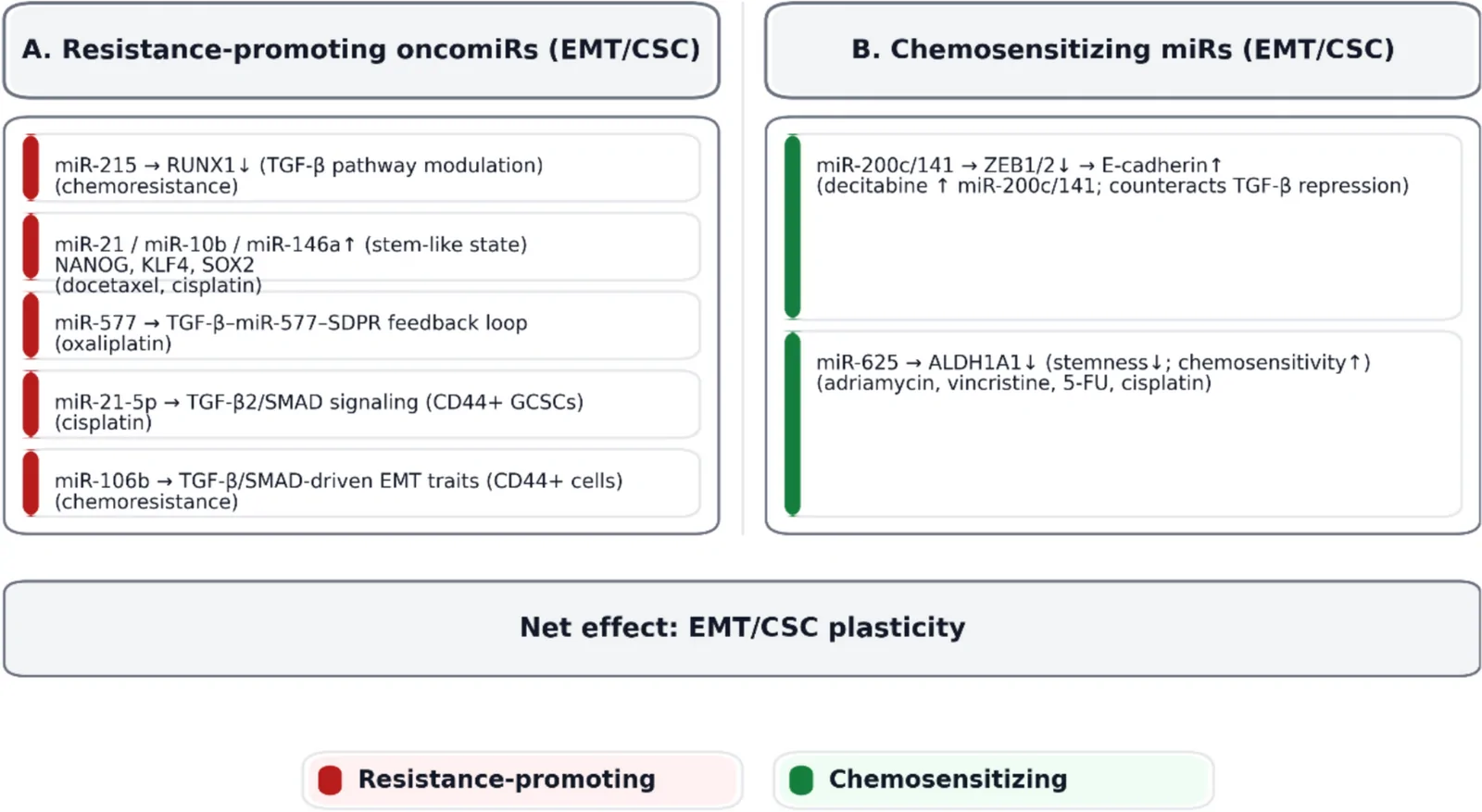
miRs involved in the regulation of EMT and the CSC phenotype. OncomiRs that promotes resistance are depicted in red, whereas chemosensitizing miRs are indicated in green

EMT is closely linked to the development and maintenance of cancer stem cell (CSC) phenotypes that can evade cytotoxic effects of conventional chemotherapy. Several miRs are critical regulators of EMT in GC (Fig. 4), and their dysregulation can promote resistance via this pathway:

- miR-215 may promote GC progression by targeting RUNX1, a transcription factor involved in the regulation of signaling pathways such as TGF-β, thereby potentially contributing to CR [90].
- higher level of miR‐21, miR‐10b, and miR‐146a was correlated with the stem‐like state of tumor cells, being evaluated by the expression of several stemness proteins (NANOG, KLF4, SOX2), potentially being responsible of resistance to docetaxel and cisplatin [91].
- miR-577 induces EMT and stemness-like properties, forming a feedback loop (TGF-β-miR-577-SDPR axis), thereby promoting metastasis and CR to oxaliplatin [92].
- miR-21—5p promotes cisplatin resistance in gastric cancer stem cells CD44 + by regulating the TGF-β2/SMAD signaling pathway [93].
- miR-106b expression also regulated cancer stem-like cell properties, particularly EMT characteristics, through the TGF-*β*/SMAD signaling pathway in CD44 + stem-like cells [94].
- miR-200c/141 may be a valuable biomarker and a therapeutic target in patients with GC since it inhibits migration and invasion of tumor cells by targeting ZEB-1/2 and consequently increasing the expression of E-cadherin. Moreover, DNA methylation and TGF- β regulate the expression of miR-200c/141 in GC. Zhou et. al. proposed Decitabine, an agent currently used in myelodysplastic syndromes and acute lymphoblastic leukemia, which acts as a DNA methyltransferase inhibitor and has been shown to increase miR-200c/141 expression while reversing the TGF-β–induced downregulation of these miRs [95].
- miR-625 mediates the downregulation of ALDH1A1, increasing the sensitivity of several chemotherapeutic agents, such as adriamycin, vincristine, 5-fluorouracil, and cisplatin [96].

### miRs regulating autophagy

**Fig. 5:**
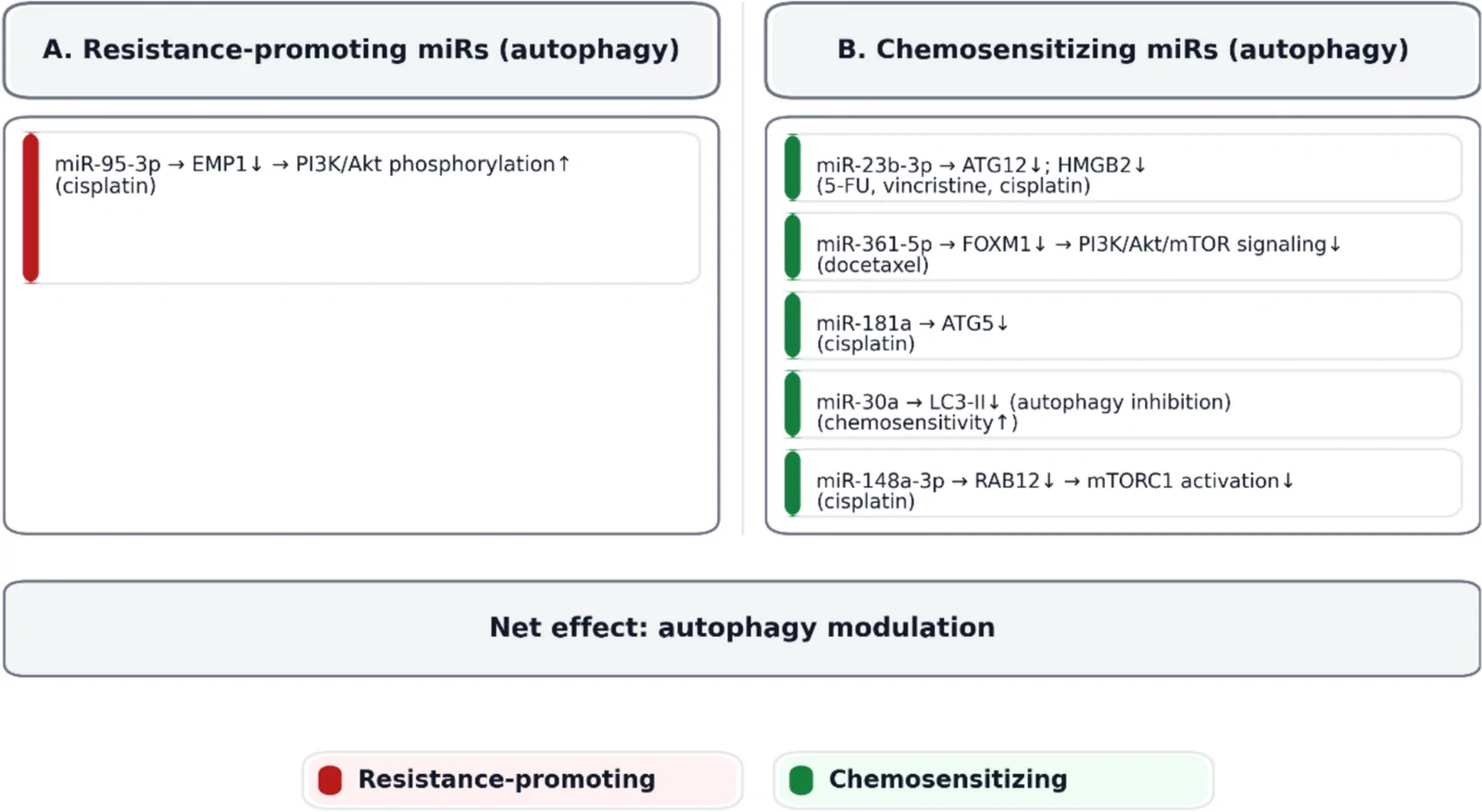
miRs regulating autophagy. Sensitizing miRs are shown in green, whereas resistance-promoting miR is indicated in red

MiRs are recognized as key regulators of autophagy in cancer, and several have been linked to CR via this pathway, as illustrated in Fig. 5.

- miR-95-3p downregulates EMP1 gene with an increasing phosphorylation of the PI3K/Akt pathway, thereby conducting to cisplatin resistance in GC [97].
- miR-23b-3p is one of the tumor-suppressive miRNAs that targets the autophagy gene ATG12 and the DNA-binding protein HMGB2, thereby sensitizing cancer cells to chemotherapeutic agents, such as 5-FU, vincristine, and cisplatin [98].
- miR-361-5p targets FOXM1 via PI3K/Akt/mTOR pathway, suppressing autophagy-induced CR to docetaxel [99].
- miR-181a, miR-30a, and miR-148a-3p have each been shown to enhance chemotherapy sensitivity by suppressing key autophagy components. For instance, miR-181a reverses cisplatin resistance by targeting ATG5 [100], miR-30a overexpression may inhibit CR-associated autophagy by reducing the LC3-II in tumor cells [101], and miR148a-3p sensitizes cancer cells to cisplatin by suppressing RAB12 expression and mTOR1 activation [102].

### miRs influencing drug metabolism and inactivation

**Fig. 6:**
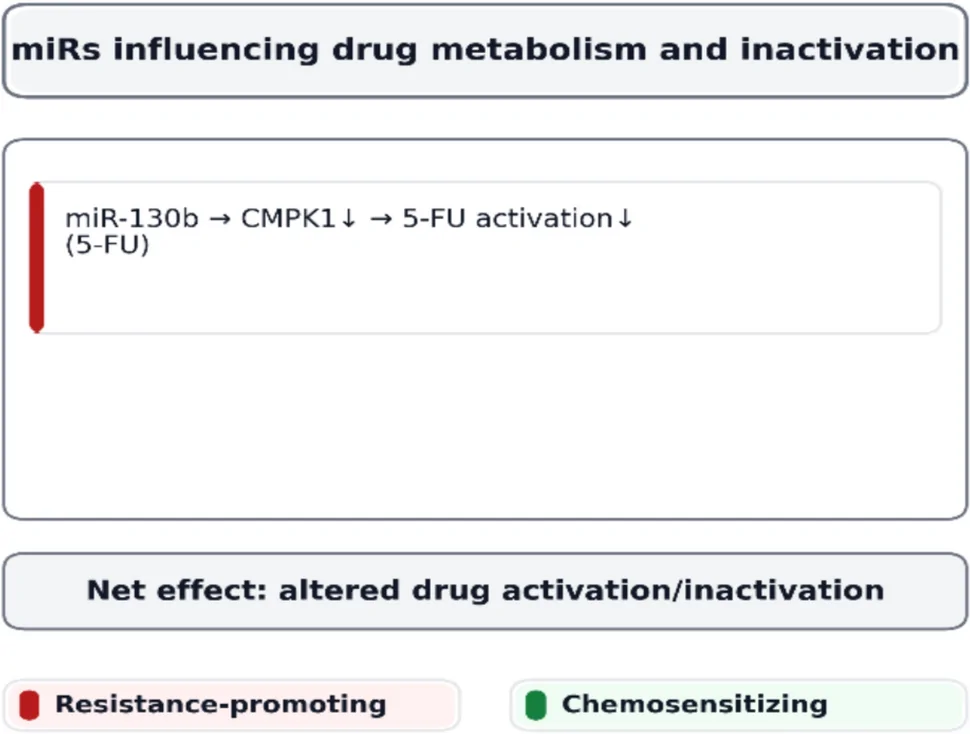
Resistance-promoting miR influencing drug metabolism is illustrated in red

Emerging evidence has revealed that miRs are key post-transcriptional regulators of genes encoding drug-metabolizing enzymes, thereby modulating the activation or inactivation of anticancer agents (Fig. 6):

- UMP/CMP kinase plays a crucial role in the conversion to the active metabolite of 5-FU. A key regulator of CMPK1 is the oncogenic miR-130b, which appears to downregulate this enzyme, resulting in resistance to 5-FU treatment [103].

### miRs altering expression or function of molecular targets

**Fig. 7:**
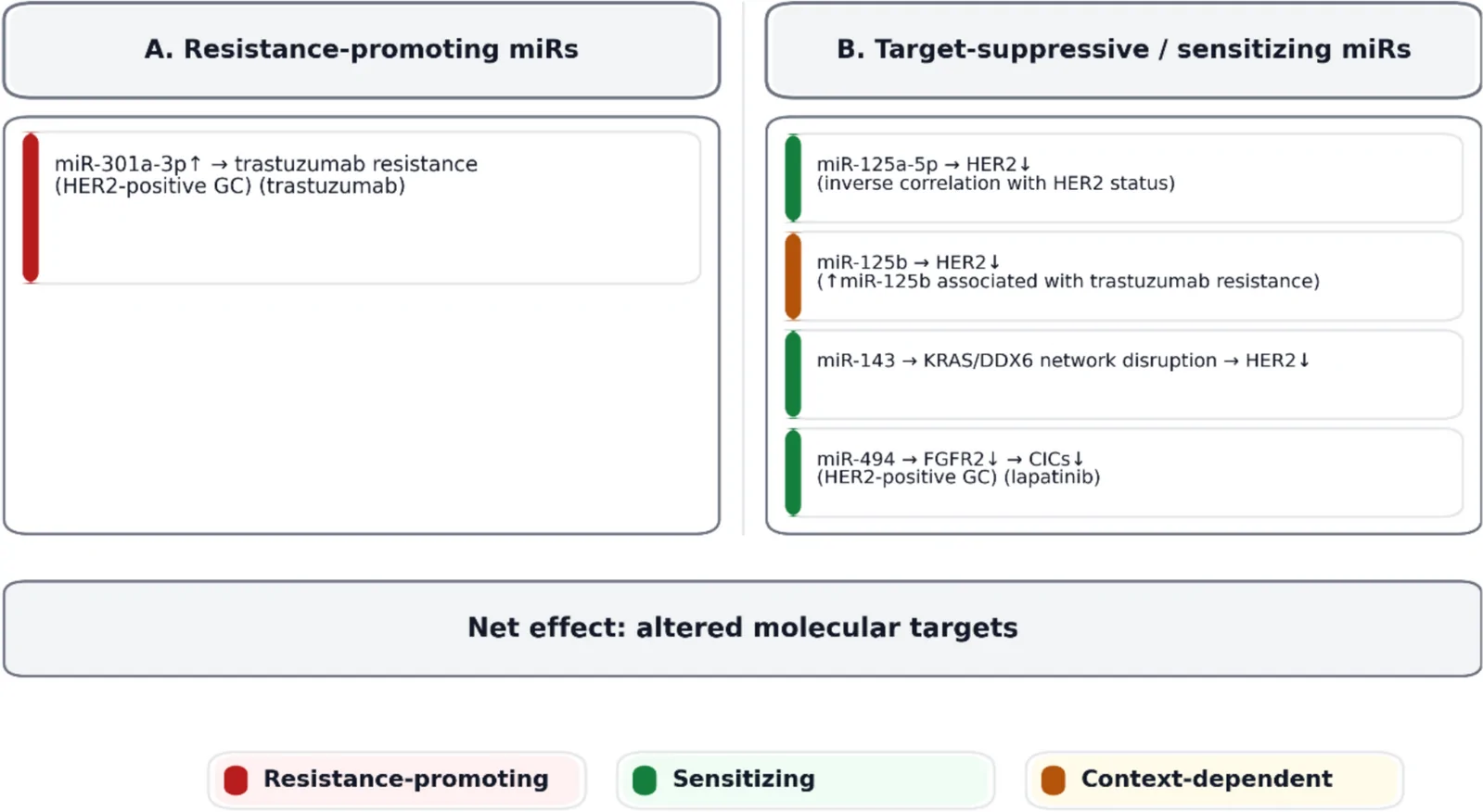
miRs altering expression/function of molecular targets. Resistance-promoting miR is shown in red, chemosensitizing miRs are indicated in green, whereas context-dependent miR is illustrated in yellow

Several miRs are playing a role in CR by directly altering the expression or function of key molecular targets, as depicted in Fig. 7:

- miR-125a-5p/125b correlates inversely with HER2 status and could be considered as therapeutic targets in GC [104]. However, miR-125b upregulation was associated with trastuzumab resistance in HER2-positive GC, potentially becoming a biomarker for predicting prognoses and clinical outcomes [105].
- Expression of miR-143 indirectly downregulates the expression of HER2 through the impairment of KRAS networks including the DDX6, acting as a tumor suppressor in GC [106].
- Overexpression of miR-494 downregulates the expression of FGFR2, inhibiting the formation of CICs and reversing lapatinib resistance in HER2-positive tumor cells [107].
- Trastuzumab resistance may be induced by the up-regulation of miR-301a-3p in HER2-positive cancer cells, potentially serving as a biomarker or a therapeutic target to overcome it [108].

### Interaction of the tumor cell with the microenvironment and the role of exosomal miRs in chemoresistance

The tumor microenvironment (TME) refers to the complex and dynamic ecosystem that surrounds and interacts with tumor cells within a solid tumor. It is composed of a diverse array of non-malignant components, including cellular elements (fibroblasts, endothelial cells, blood vessels, immune cells—macrophages, T cells, dendritic cells), extracellular matrix (ECM) and non-cellular components (exosomes and cytokines). These elements do not merely serve as passive bystanders; rather, they engage in reciprocal interactions with tumor cells, influencing key processes, such as tumor growth, invasion, metastasis, angiogenesis, immune evasion, and therapeutic resistance [109]. An important conceptual advance in recent years is the recognition that miR dysregulation within the tumor microenvironment is not limited to tumor cells themselves. Stromal and immune compartments—including cancer-associated fibroblasts, macrophages, endothelial cells, and extracellular vesicles—can all contribute functionally relevant miRs that reshape treatment response [11].

Hypoxia represents a common feature of the TME. There are several hypoxic factors prevalent in most solid tumors that provide a dysfunctional vascular system. Carcinogenic factors, such as drug, carcinogen, and microbiota dysbiosis, TME remodeling resulting in vascular deformation, high metabolism present in cancer cells and a non-functional angiogenesis are among them [110].

**Fig. 8:**
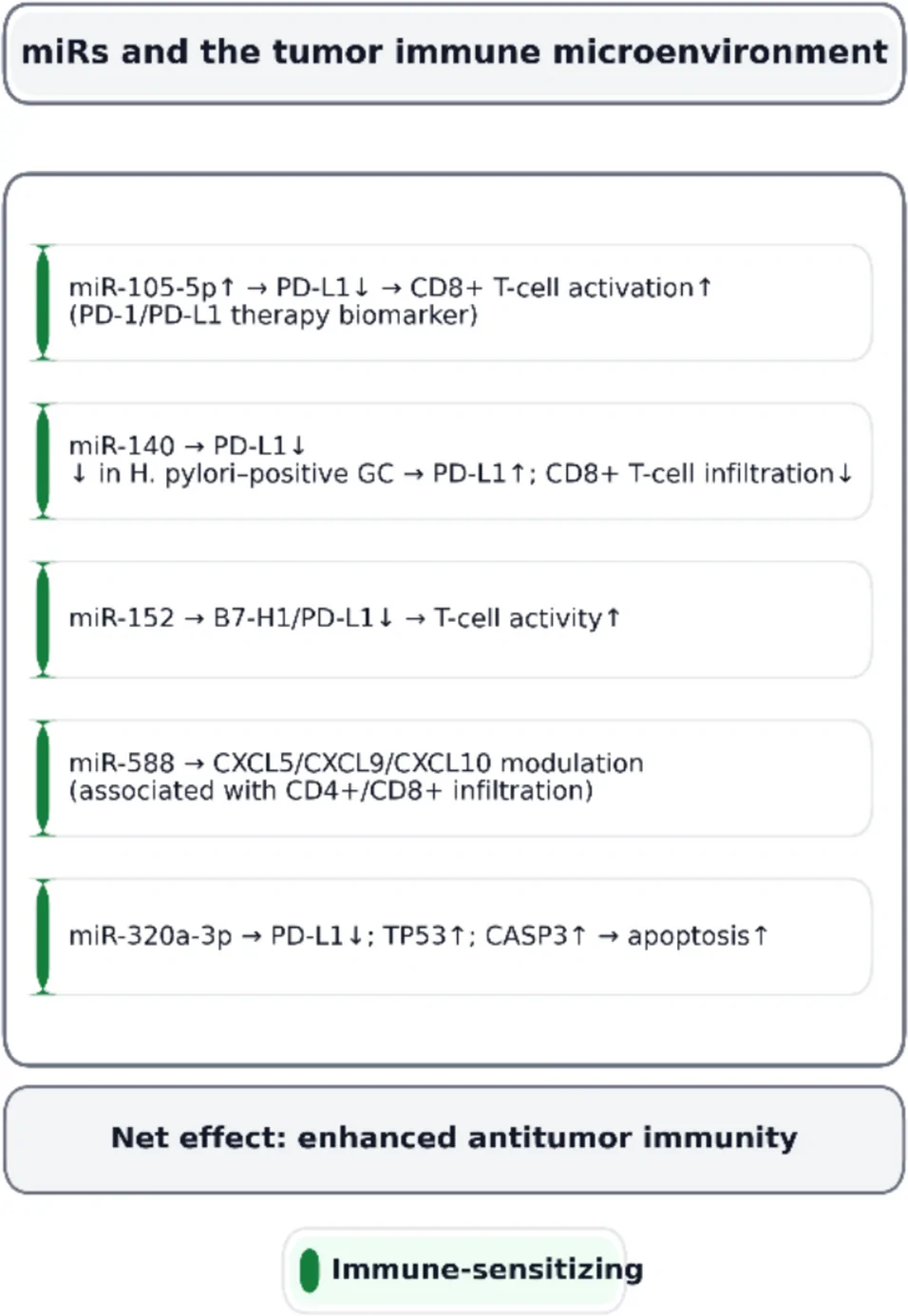
Sensitizing miRs influencing the TME are indicated in green

Immune evasion could also be promoted due to immunosuppressive changes in the TME, which may limit the effectiveness of immune checkpoint inhibitors (ICI)—nivolumab and pembrolizumab, even in PD-L1-positive tumors. The main predictive biomarkers for ICI are microsatellite instability, which has the strongest value among them, tumor mutational burden and PD-L1 expression [111]. Representative miRNAs influencing the tumor immune microenvironment, as shown in Fig. 8, include:

- Overexpression of miR-105-5p in tumor cells resulted in decreased expression of PD-L1 and in CD8 + T cell activation, controlling their immunogenicity. Thereby, it could be a potential biomarker for PD-1/PD-L1 therapy [112].
- miR-140 acts as a tumor suppressor in GC by directly targeting PD-L1. Its downregulation in *Helicobacter pylori*-positive cases is associated with increased PD-L1 expression, enhanced tumor cell proliferation, and an immunosuppressive microenvironment characterized by reduced CD8⁺ T cell infiltration [113].
- miR-152 acts as a tumor suppressor via targeting and inhibiting the 3’UTR of B7-H1(PD-L1), thereby enhancing T cells proliferation and function [114].
- miR-588 targets CXCL5, CXCL9, and CXCL10 genes, correlating positively with infiltrating levels of CD4 + and CD8 + T cells, and may be a biomarker for tumor-associated immune infiltration [115].
- miR-320a-3p could suppress PD-L1 gene expression and induce apoptosis by increasing TP53 and CASP3, potentially being a target in GC immunotherapy [116].
- miR-1290 acts as an oncomiR by up-regulating PD-L1 via the GRHL2/ZEB1 pathway and promoting the suppressive action of GC cells on T cell activation [117].

**Fig. 9:**
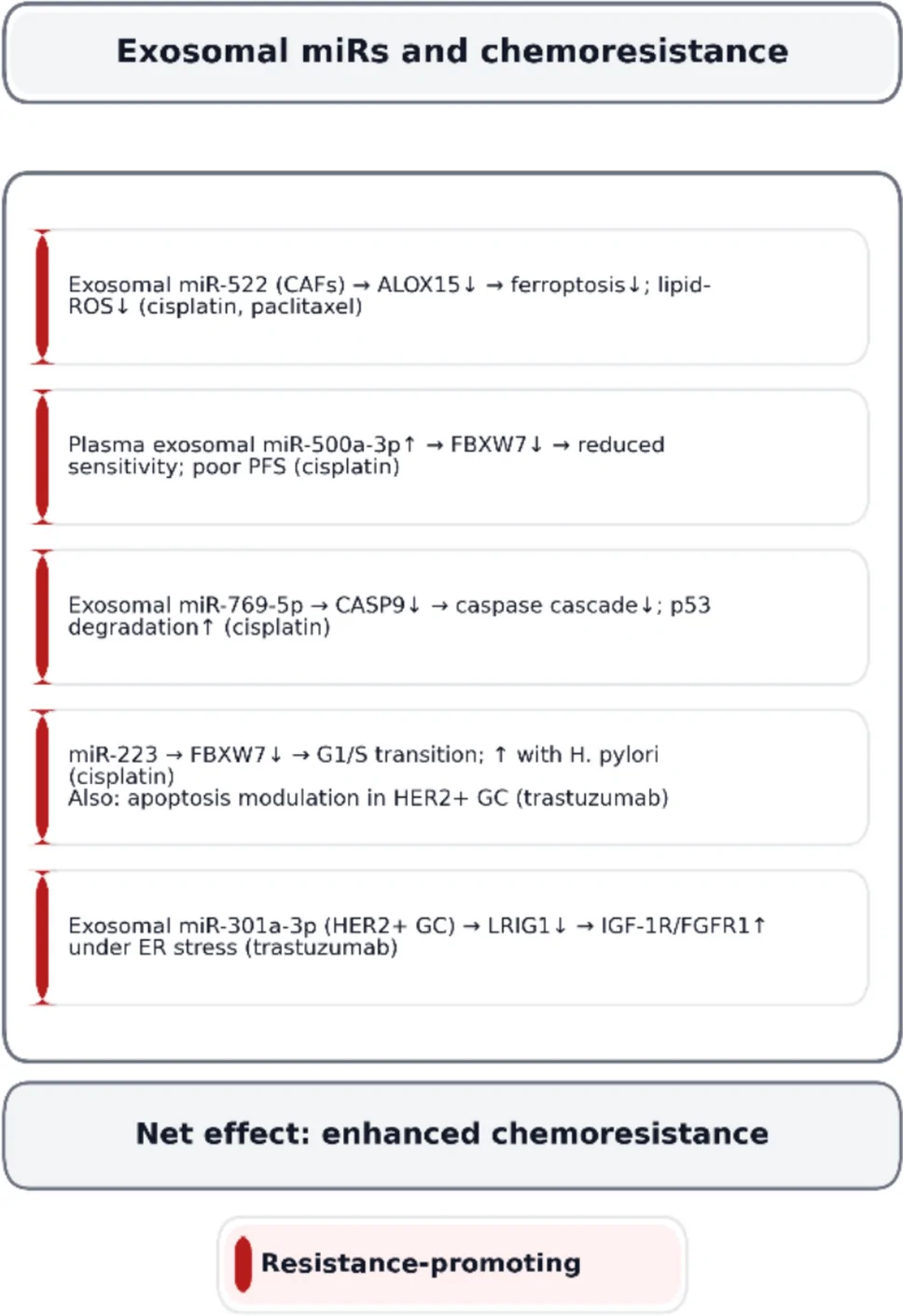
Resistance-promoting miRs via exosomes are shown in red

Exosomal miRs allow stromal cells like cancer-associated fibroblasts (CAFs) to communicate pro-survival signals to cancer cells, thereby modulating drug response [118]. Examples of intercellular transfer of miRNAs via exosomes, as depicted in Fig. 9, include:

- Exosome-miR-522 secreted by CAFs inhibits ferroptosis by targeting ALOX15 and blocking lipid-ROS accumulation, contributing to a decreased sensitivity to cisplatin and paclitaxel [119].
- Elevated miR-500a-3p in the plasma exosomes of patients with GC induce cisplatin resistance through targeting FBXW7, resulting in poor progression-free prognosis, and could potentially predict CR in GC patients [120].
- Exosomal miR-769-5p contributes to cisplatin resistance by targeting CASP9, thereby suppressing activation caspase cascade and facilitating ubiquitin–proteasome-mediated degradation of the pro-apoptotic protein p53 [121].
- miR-223 directly targets FBXW7 gene and could affect G_1_/S transition of cell cycle, promoting cisplatin resistance in tumor cells. Additionally, miR-223 is significantly up-regulated in *H*. *pylori* infection, suggesting that it may lead to GC CR [122]. The miR-223/FBXW7 pathway modulates the sensitivity of HER2-positive GC cells to trastuzumab by regulating apoptotic signaling, suggesting a potential mechanism of trastuzumab resistance [123].
- Exosomal miR-301a-3p in HER2-positive tumor cells could mediate trastuzumab resistance by down-regulating LRIG1 and subsequently activating the expression of IGF-1R and FGFR1 under endoplasmic reticulum stress [124].

Taken together, these findings highlight the potential of miR profiling as a valuable tool for predicting chemotherapy response and guiding therapeutic strategies as well as developing novel therapeutic agents, including immunotherapy.

## Conclusion

MicroRNAs have emerged as key regulators of CR in GC, acting at multiple levels of the drug–tumor interaction. Through the modulation of pathways involved in drug transport, DNA damage repair, apoptosis, epithelial–mesenchymal transition, autophagy, and immune escape, miRs function as upstream network regulators that coordinate complex resistance programs rather than single molecular targets. Their role extends beyond tumor-intrinsic signaling to include interactions with the tumor microenvironment, where stromal cells and exosome-mediated communication further reinforce adaptive survival and therapy resistance.

Beyond their mechanistic relevance, miRs are increasingly recognized as important components of precision oncology in GC. Detectable in tumor tissue, plasma, and extracellular vesicles, they represent promising biomarkers for predicting treatment response and monitoring the emergence of resistance during therapy. At the same time, their upstream regulatory role offers opportunities for therapeutic intervention, as restoring tumor-suppressive miRs or inhibiting oncogenic miRs may simultaneously affect multiple resistance pathways.

However, translating these findings into clinical practice requires overcoming several challenges, including the standardization of sampling and analytical methods, validation of robust miRs panels in large prospective cohorts, and the development of efficient and tumor-selective delivery systems for miR-based therapies.

Future research should focus on integrating miR profiling into clinical decision-making frameworks, improving delivery platforms for miR-based therapeutics, and exploring combination strategies with chemotherapy, targeted therapies, and immunotherapy. Such approaches may ultimately enable more precise patient stratification and the development of personalized treatment strategies capable of overcoming CR in GC.

## Data Availability

The data supporting the findings of this study are available from the corresponding author upon reasonable request.

## Declarations

### Generative AI and AI-assisted technologies in the writing process

During the preparation of this work the author(s) used OpenAI. (2026, February 15). ChatGPT (Version 5) [Large language model]. OpenAI. https://openai.com in order to improve the language and readability. After using this tool/service, the author(s) reviewed and edited the content as needed and take(s) full responsibility for the content of the publication.
